# MRI‐Based Human Study on the Neuroprotective Effects of Probiotic KLP301 in subjects with Mild Cognitive Impairment

**DOI:** 10.1002/alz70861_108726

**Published:** 2025-12-23

**Authors:** Taemin Kim

**Affiliations:** ^1^ Chosun university, Gwangju, Gwangju Korea, Republic of (South)

## Abstract

**Background:**

Alterations in the gut microbiota can influence the onset and progression of Alzheimer's disease through the gut‐brain axis. Dysbiosis may trigger inflammation and immune dysfunction, contributing to key Alzheimer’s disease pathologies. Recent studies suggest that probiotics may help delay cognitive decline and reduce inflammation.

**Method:**

In our previous study, we selected KLP301, a multi‐strain probiotic culture, based on its antioxidant activity, anti‐inflammatory effects, and intestinal colonization ability. In this human study, 20 participants were assigned to the experimental group and received KLP301 for 52 weeks, while another 20 participants in the control group received maltodextrin for the same period. Brain structural images were obtained using MRI at baseline and after 52 weeks of administration. Changes in brain structure between the two time points were compared and analyzed between the control and experimental groups.

**Result:**

The group administered KLP301 showed a smaller reduction in cortical thickness change in the left isthmus of the cingulate gyrus and the right cuneus compared to the control group. The decrease in subcortical volume change in the right caudate was also less pronounced in the KLP301 group. Furthermore, the KLP301 group exhibited a significantly attenuated reduction in cortical surface area change in the left lateral orbitofrontal cortex, right rostral middle frontal gyrus, left parahippocampal gyrus, and right parahippocampal gyrus compared to the control group.

**Conclusion:**

KLP301 multi‐strain probiotic culture may suppress brain atrophy in subjects with MCI, highlighting its potential as a health functional food for the prevention and management of Alzheimer's disease.

